# Controlling selectivities in CO_2_ reduction through mechanistic understanding

**DOI:** 10.1038/s41467-017-00558-9

**Published:** 2017-09-11

**Authors:** Xiang Wang, Hui Shi, János Szanyi

**Affiliations:** Institute for Integrated Catalysis, Pacific Northwest National Laboratory, Richland, WA 99352 USA

## Abstract

Catalytic CO_2_ conversion to energy carriers and intermediates is of utmost importance to energy and environmental goals. However, the lack of fundamental understanding of the reaction mechanism renders designing a selective catalyst inefficient. Here we show the correlation between the kinetics of product formation and those of surface species conversion during CO_2_ reduction over Pd/Al_2_O_3_ catalysts. The *operando* transmission FTIR/SSITKA (Fourier transform infrared spectroscopy/steady-state isotopic transient kinetic analysis) experiments demonstrates that the rate-determining step for CO formation is the conversion of adsorbed formate, whereas that for CH_4_ formation is the hydrogenation of adsorbed carbonyl. The balance of the hydrogenation kinetics between adsorbed formates and carbonyls governs the selectivities to CH_4_ and CO. We apply this knowledge to the catalyst design and achieve high selectivities to desired products.

## Abstract (web summary)

Understanding the mechanism of CO_2_ reduction on a catalyst surface is essential for achieving the desired product selectivity. Here, the authors show an *operando* kinetic analysis of CO_2_ hydrogenation over a palladium catalyst in order to address the factors governing the selectivity of the process.

## Introduction

Heterogeneous catalytic CO_2_ reduction has been attracting great attention, because it not only mitigates the risks associated with increasing CO_2_ concentration in the atmosphere but also offers a variety of pathways for producing fuels and industrial chemicals^1, 2^. Under atmospheric pressure, methanation and reverse water gas shift (RWGS) reaction are the two pathways for thermocatalytic CO_2_ reduction on Group VIII metals^2^. Although methanation produces synthetic natural gas, RWGS generates CO, an important component of syngas. Considerable efforts have been devoted to developing catalysts to achieve high selectivity towards either CH_4_ or CO^3–9^. However, for designing a selective catalyst, it is very important to first have a fundamental understanding of the site requirements, elementary steps and intermediates, which can ultimately lead to the construction of viable mechanisms for both methanation and RWGS. Although intricate interplay between various surface species at different catalyst components that determine product selectivity has been recognized, controversies still exist regarding the detailed mechanism and the roles of surface species^10–15^. Some issues may originate from the different interrogation methods used; for example, some prior conclusions were based on kinetic and spectroscopic information acquired under transient conditions^12^. Under transient conditions, the perturbation of the chemical environment around the adsorbates and catalyst surface causes characteristically divergent behaviors from steady state.

Steady-state isotopic transient kinetic analysis (SSITKA) has evolved as one of the most powerful techniques allowing measurements at steady state to determine the mean surface lifetimes and abundances of intermediates leading to products^16, 17^. However, SSITKA itself cannot identify the chemical nature of surface species, a task that can be accomplished by *operando* spectroscopic measurements, e.g., Fourier transform infrared spectroscopy (FTIR). Correlating the reaction kinetics with the transformations of spectroscopically observable surface species, both simultaneously measured under steady-state reaction conditions, is vital for the complete elucidation of the complex mechanistic framework^18–21^. Pd-based catalysts are able to catalyze both CO_2_ methanation and RWGS^22, 23^. Our previous work on Pd/Al_2_O_3_ bifunctional catalysts has proposed the plausible pathways for both CO_2_ methanation and RWGS^24^. However, the factors that govern product selectivity (to CH_4_/CO) under steady-state CO_2_ reduction conditions are still not clear.

Here we show that CH_4_ and CO selectivities are governed by the balance of the hydrogenation kinetics between adsorbed formates and carbonyls over Pd/Al_2_O_3_ catalysts, which was demonstrated by a combined *operando* transmission FTIR spectroscopy/SSITKA investigation. Using this knowledge, we show that tailor-made catalysts can be prepared to achieve high selectivity to either of the two desired products.

## Results

### Steady-state isotopic transient kinetic analysis

**Fig. 1:**
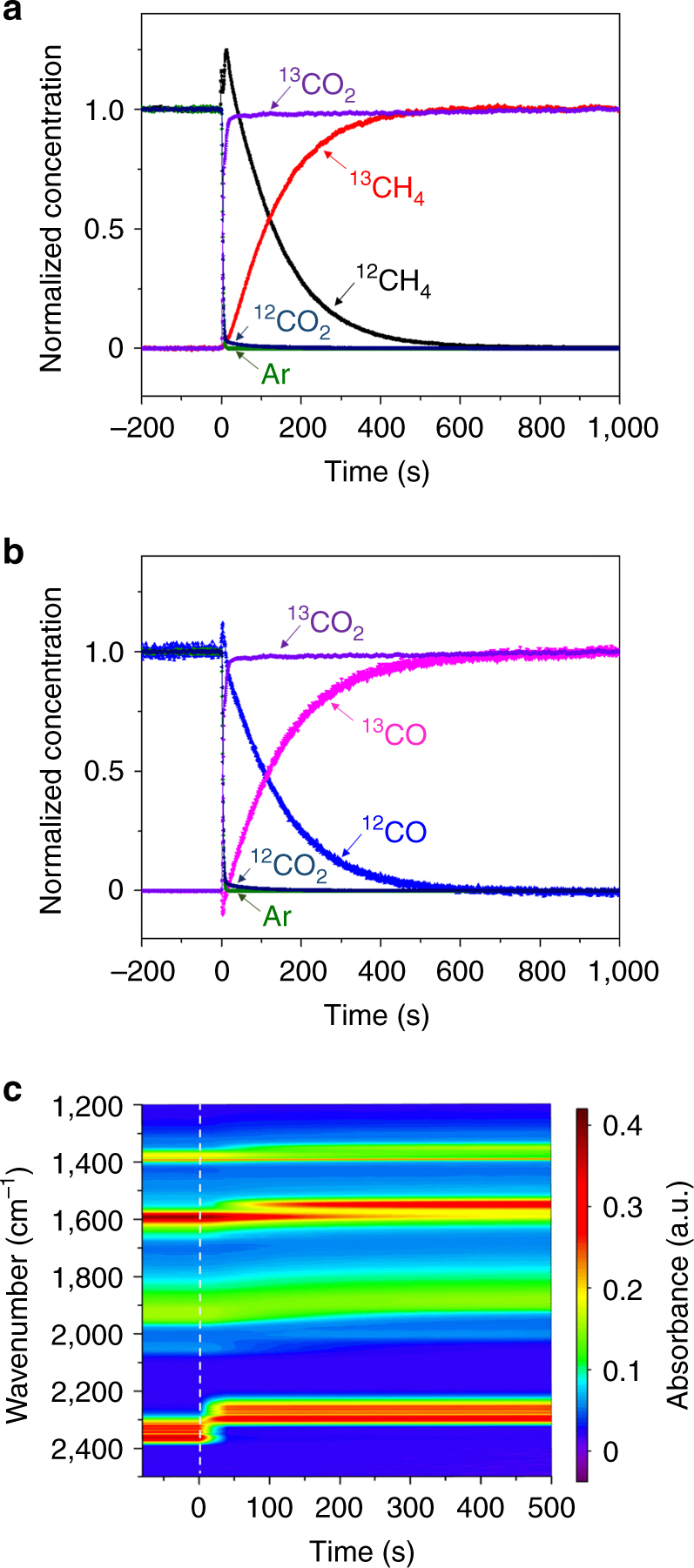
The responses of reactants, products and surface intermediates after the steady-state isotopic switch. **a**, **b** Normalized mass spectrometry signals of gases in the effluent and **c** FTIR contour plot collected as a function of time. The feed gas was switched at 0 s from ^12^CO_2_/H_2_/Ar to ^13^CO_2_/H_2_ over 5% Pd/Al_2_O_3_ at 533 K. Ar gas was used as an inert tracer

Figure 1a, b show normalized mass spectrometry (MS) responses of gases in the effluent after the feed gas was switched at 0 s from ^12^CO_2_/H_2_/Ar to ^13^CO_2_/H_2_ at 533 K. The fast disappearance of Ar gas in <4 s indicates a short gas hold-up time for the system. The disappearance of ^12^CO_2_ was almost as fast as Ar, indicating weak interaction between ^12^CO_2_ and the catalyst and the reactor walls. In the meantime, the concentration of ^13^CO_2_ signal increased accordingly, with the concentration and the conversion (2.5%) of carbon dioxide (^12^CO_2_ + ^13^CO_2_) constant during the switch. The decaying signals of ^12^CH_4_ and ^12^CO and the rising signals of ^13^CH_4_ and ^13^CO crossed at *y* = 0.5, indicating constant rates for methane and carbon monoxide formation regardless of isotopic substitution. These symmetrical responses indicate that the steady state of the catalyst surface was not perturbed during the switch. The concentrations of products ^12^CH_4_ and ^12^CO decreased more slowly than that of ^12^CO_2_ and disappeared at ~600 s. This indicates large mean residence times for C-containing surface intermediates leading to the products, and that intermediates which equilibrate with CO_2_ are not involved in the rate-determining steps for CO_2_ methanation and RWGS.

Figure 1c shows the corresponding *operando* FTIR contour plot. Before the switch, the absorption band between 2,300 and 2,400 cm^−1^ (red range) is attributed to gaseous ^12^CO_2_. The spectral range between 1,800 and 2,100 cm^−1^ (blue and green range) is assigned to chemisorbed carbonyls (^∗^^12^CO) on the surface of Pd metal particles^22^. The absorption bands around 1,400 (yellow range) and 1,600 cm^−1^ (red range) arise from adsorbed formates (H^12^COO^∗^) on the surface of the Al_2_O_3_ support^22^. After the switch, the bands due to ^12^CO_2_ quickly disappeared and a new band developed at lower wavenumbers (red range between 2,200 and 2,300 cm^−1^) attributable to ^13^CO_2_, consistent with their rapid responses in MS. The IR signal decay of surface species might be slightly affected by the location of IR beam on the sample wafer^25^. However, the shift of the IR features of ^12^CO^∗^ and H^12^COO^∗^ to those of ^13^CO^∗^ and H^13^COO^∗^ at lower wavenumbers were much slower compared with the shift of ^12^CO_2_ to ^13^CO_2_ bands. This observation suggests that neither H^12^COO^∗^ nor ^12^CO^∗^ undergoes reverse reactions to form ^12^CO_2_. The disappearance trends of the IR signatures of H^12^COO^∗^ and ^12^CO^∗^ are similar to those of the MS signals of ^12^CH_4_ and ^12^CO products, suggesting that both H^12^COO^∗^ and ^12^CO^∗^ are reactive intermediates rather than spectators and, the rate-determining steps for CH_4_ and CO formation are related to CO^∗^ and HCOO^∗^. It is noteworthy that no other species (e.g., bicarbonate) were observed under steady-state reaction conditions and no carbon deposition was identified in our previous study on Pd/Al_2_O_3_ during CO_2_ reduction^24^. Therefore, CO^∗^ and HCOO^∗^ are the two most abundant surface species and will be the focus of the following discussion.

To gain insight into the interaction between ^∗^CO and Pd on the 5% Pd/Al_2_O_3_, room temperature ^12^CO adsorption followed by temperature-programed ^∗^^12^CO desorption were studied by FTIR (Supplementary Fig. 1). At room temperature ^∗^^12^CO on Pd showed IR features at 2,098, 2,052, 1,960 and 1,928 cm^−1^. When the temperature was raised to 533 K, ^∗^^12^CO with features at 2,098 and 1,960 cm^−1^ desorbed, whereas ^∗^^12^CO with bands at 2,052 and 1,928 cm^−1^ did not desorb but was hydrogenated to CH_4_ once H_2_ was introduced. This reveals the existence of two types of adsorption sites on the Pd particles that bind CO weakly (^∗^^12^CO_w_) and strongly (^∗^^12^CO_s_). The stability of ^∗^CO_s_ at high temperatures is due to the dissociated ^∗^H, which adsorbs near to ^∗^CO_s_ on the Pd surface and transfers electron density to Pd to strengthen the Pd-CO bond^22, 26^. The FTIR spectrum of ^∗^^12^CO_s_ is very similar to that of ^∗^^12^CO obtained in CO_2_ reduction at 533 K, indicating that all ^∗^^12^CO observed under reaction conditions were ^∗^^12^CO_s_. However, ^∗^^12^CO_w_ could not be observed during CO_2_ reduction at ≥ 533 K due to its rapid desorption. As we observe no ^∗^^12^CO_w_ under steady-state reaction conditions, the ^12^CO product generated after the switch was mainly from the conversion of the other abundant species, H^12^COO^∗^.

Normalized MS responses of ^12^CH_4_ and ^12^CO with increasing temperature are shown in Supplementary Fig. 2, whereas those for ^13^CH_4_ and ^13^CO are not displayed. The faster decay in both ^12^CH_4_ and ^12^CO signals at higher temperatures indicates the higher reactivities of intermediates. In contrast, the decay curves of ^12^CO_2_ and Ar did not change with temperature, further indicating that any elementary step that directly involves CO_2_ is not the rate-limiting step for the formation of CH_4_ and CO. The real mean surface residence times ${{{\bar \tau }}_{{0}}}$ (Supplementary Fig. 3) of intermediates leading to ^12^CH_4_ and ^12^CO (denoted as ICH and ICO) together with the ${{{\bar \tau }}_{{0}}}$-derived activation energies *E* for ^12^CH_4_ and ^12^CO formation (Supplementary Fig. 4) are summarized in Table 1. The ${{{ \bar \tau }}_{{{0}}\_{{\rm ICH}}}}$ at 533 K was 134 s and gradually decreases with increasing temperature, reaching 41 s at 573 K. Similarly, the ${{{ \bar \tau }}_{{{0}}\_{{\rm ICO}}}}$ decreased from 107 to 55 s with increasing temperature from 533 to 573 K. The activation energies are 75 and 53 kJ mol^−1^ for the formation of CH_4_ and CO, respectively, in line with the values we obtained previously in a plug-flow reactor^24^. Series of corresponding *operando* FTIR spectra of ^∗^CO and HCOO^∗^ collected at 533–573 K are shown in Supplementary Figs. 5, 6, respectively. The shift of IR bands representing ^∗^^12^CO_s_ and H^12^COO^∗^ to those representing ^∗^^13^CO_s_ and H^13^COO^∗^ became faster with increasing temperature, but still remained much slower than the shift of the IR band of ^12^CO_2_ to that of ^13^CO_2_. The decay trends of ^∗^^12^CO_s_ and H^12^COO^∗^ FTIR intensity with increasing temperature was very similar to the decay trends of MS signals of ^12^CH_4_ and ^12^CO products (Supplementary Fig. 2), further indicating that HCOO^∗^ and CO^∗^ are related to the rate determining steps for CO and CH_4_ formation. In addition, the disappearances of ^∗^^12^CO_s_ and H^12^COO^∗^ follow an apparent first-order kinetics. The activation energies based on the Arrhenius plots of the two first-order rate constants (Supplementary Figs. 5, 6) are listed in Table 1. The activation energy for the conversion of ^∗^^12^CO_s_ was 73 kJ mol^−1^, an identical value to that determined for ^12^CH_4_ formation from the MS data. This result suggests that the rate-determining step along the CH_4_ formation path is the conversion of ^∗^CO_s_. The activation energy of H^12^COO^∗^ conversion was 52 kJ mol^−1^, very similar to the 53 kJ mol^−1^ determined for the ^12^CO formation by MS, indicating that the conversion of HCOO^∗^ is the rate-determining step for CO formation.

**Table 1:** Real mean surface residence times for ICH $\left( {{{{{ \bar \tau }}}_{{{0}}\_{{\rm ICH}}}}} \right)$ and ICO $\left( {{{{{ \bar \tau }}}_{{{0}}\_{{\rm ICO}}}}} \right)$, and activation energies for CH_4_ (*E*_CH4_) and CO (*E*_CO_) formation and adsorbed ∗COs (*E*∗COs) and HCOO^∗^ (*E*_HCOO_∗) conversions in CO_2_ reduction at 533–573 K

**Temp.** **(K)**	${{{ \bar \tau }}_{{\bf{0}}\_{\bf{ICH}}}}$ **(s)**	${{{ \bar \tau }}_{{\bf{0}}\_{\bf{ICO}}}}$ **(s)**	***E***_**CH4**_ (**kJ** **mol** ^***−1***^)	***E***_**CO**_ **(kJ** **mol** ^***−1***^)	**E**∗_COs_ **(kJ** **mol** ^***−1***^)	**E**_**HCOO**_∗**(kJ** **mol** ^***−1***^)
533	134	107				
543	92	85	75	53	73	52
563	49	57				
573	41	55				

Uncertainties in activation energies are ±2 kJ mol^−1^

### Abundance of surface intermediates

**Fig. 2:**
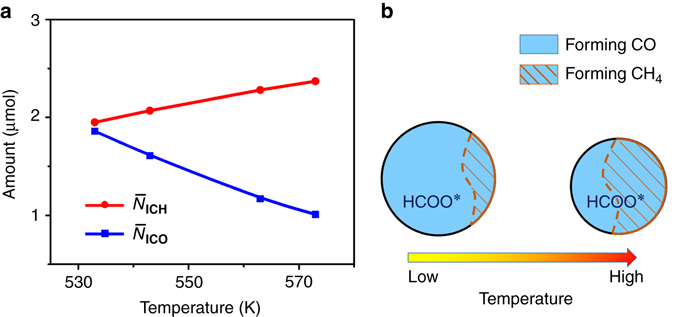
The distribution of surface intermediates in the pathways under steady state. **a** The amount of surface intermediates leading to ^12^CH_4_ $\left( {{{{{\bar N}}}_{{{\rm ICH}}}}} \right)$ and ^12^CO $\left( {{{{{ \bar N}}}_{{{\rm ICO}}}}} \right)$ on the catalyst (41 mg) during CO_2_ reduction at 533–573 K. **b** Schematic representation of the proportion of surface coverage of HCOO^∗^ that is eventually converted to CH_4_ and CO with increasing temperature concluded from **a**

Figure 2 shows the surface abundance $\left( {{{ \bar N}}} \right)$ of ICH and ICO from SSITKA at 533–573 K. The ${{{ \bar N}}_{{{\rm ICO}}}}$ was 1.86 μmol at 533 K, and decreased to 1.01 μmol as the temperature was increased to 573 K. As demonstrated above, ^12^CO was produced from the existing H^12^COO^∗^. Thus, the amount of H^12^COO^∗^ that was converted to ^12^CO was less at higher temperatures than at lower temperatures. In contrast, the ${{{ \bar N}}_{{{\rm ICH}}}}$ increased from 1.95 μmol at 533 K to 2.37 μmol at 573 K. This increase could be interpreted as being caused by an increased concentration of ^∗^^12^CO_s_ at higher temperatures. However, this is not the case, as no difference in intensity of ^∗^^12^CO_s_ was observed as the temperature was increased from 533 to 573 K (Supplementary Fig. 7). The constant intensity of ^∗^^12^CO_s_ implies that the amount of ^12^CH_4_ produced from ^∗^^12^CO_s_, which have already existed at the moment of the switch, should be the same at 533–573 K. Therefore, the significant increase in the amount of ^12^CH_4_ at higher temperatures is attributed to the conversion of the other abundant species, H^12^COO^∗^. These results suggest that with increasing temperature, for a given amount of surface HCOO^∗^ at steady state, a progressively larger portion enters the CO_2_ methanation pathway, while a smaller portion enters the RWGS pathway (Fig. 2b). This explains the decreased ${{{ \bar N}}_{{{\rm ICO}}}}$ at higher temperature. This also agrees well with the above SSITKA results, which show that the ${{{ \bar \tau }}_{{{0}}\_{{\rm ICH}}}}$ became shorter than ${{{ \bar \tau }}_{{{0}}\_{{\rm ICO}}}}$ with increasing temperature. Therefore, formates prefer to enter the faster pathway to form CH_4_ rather than enter the slower pathway to form CO at higher temperature. Similarly, Goodwin et al. discussed a case where two products share an intermediate: if ${{{ \bar \tau }}_{{1}}}$ > ${{{ \bar \tau }}_{{2}}}$, then it must be that ${{{ \bar N}}_{{1}}}$<${{{ \bar N}}_{{2}}}$, because more of this intermediate will form product 2 than product 1 due to the faster formation of product 2 (smaller ${{{ \bar \tau }}_{{2}}}$)^27^. Therefore, the temperature effect on these parameters, ${{{ \bar \tau }}_{{{0}}\_{{\rm ICH}}}}$, ${{{ \bar \tau }}_{{{0}}\_{{\rm ICO}}}}$, ${{{ \bar N}}_{{{\rm ICH}}}}$ and ${{{\bar N}}_{{{\rm ICO}}}}$, further demonstrates that HCOO^∗^ is the intermediate shared by the processes of both CO_2_ methanation and RWGS.

## Discussion

**Fig. 3:**
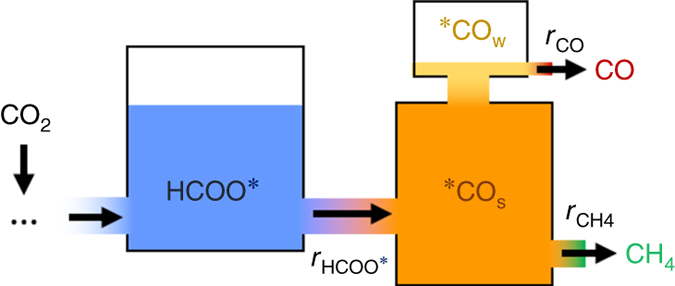
Factors governing the selectivities in CO_2_ reduction. Scheme for the pathways from formate to products in CO_2_ methanation and RWGS over Pd/Al_2_O_3_ catalysts. The filled pool of ^∗^CO_s_ and the unfilled pool of HCOO^∗^ are supported by the unchanged coverage of ^∗^^12^CO_s_ and the changed coverage of H^12^COO^∗^ at 533–573 K (Supplementary Fig. 7)

Based on above results and SSITKA theory^16, 17^, a scheme relating HCOO^∗^, ^∗^CO_s_ and ^∗^CO_w_ species in CO_2_ reduction is proposed in Fig. 3. The reduction of formate produces ^∗^CO that first occupies strong adsorption sites on the Pd particles. Once the strong adsorption sites of Pd particles are saturated (the pool of ^∗^CO_s_ is completely filled), the excess ^∗^CO can only occupy the weak adsorption sites of Pd particles to form ^∗^CO_w_. The ^∗^CO_s_ will be further hydrogenated to CH_4_ while the ^∗^CO_w_ will desorb.

The scheme shows that the rate of CO formation (*r*_CO_) is determined and equal to the rate of HCOO^∗^ conversion (${r_{{\rm{HCOO}}^{*}}}$) subtracted by the rate of CH_4_ formation (*r*_CH4_):

1 $${r_{{\rm{CO}}}} = {r_{{\rm{HCOO}}^{*}}} - {r_{{\rm{CH4}}}}$$

The rate of CH_4_ formation is equal to the rate of ^∗^CO_s_ conversion (${r_{{\,}^{*}{\rm{COs}}}}$, the rate-determining step for CH_4_ formation), which is proportional to the concentration of ^∗^CO_s_. The completely filled pool of ^∗^CO_s_ in the 533–573 K temperature range suggests that ${r_{{\,}^{*}{\rm{COs}}}}$ has reached its maximum, ${r_{{\,}^{*}{\rm{COs}}\_{\rm{max}}}}$, at each given temperature between 533–573 K. Therefore, it is the balance between the rate of HCOO^∗^ reduction to ^∗^CO (${r_{{\rm{HCOO}}^{*}}}$) and the maximum rate of ^∗^CO_s_ conversion (${r_{{\,}^{*}{\rm{COs}}\_{\rm{max}}}}$) that determines whether CO is observed in the gas phase or not. If ${r_{{\rm{HCOO}}^{*}}}$ > ${r_{{\,}^{*}{\rm{COs}}\_{\rm{max}}}}$, both CH_4_ and CO will be observed (e.g., the case in Fig. 3). If, however, ${r_{{\rm{HCOO}}^{*}}}$ ≤ ${r_{{\,}^{*}{\rm{COs}}\_{\rm{max}}}}$, which means that all the ^∗^CO produced from HCOO^∗^ are not sufficient to saturate all strong adsorption sites on the Pd metal for CH_4_ formation (i.e., the pool of ^∗^CO_s_ in Fig. 3 is not full or just full), then all ^∗^CO will be ^∗^CO_s_ and will be further hydrogenated to CH_4_. In this case, CO_2_ reduction will only be methanation and the rate of CH_4_ formation *r*_CH4_ (${r_{{\,}^{*}{\rm{COs}}}}$) will not reach its maximum${r_{{\,}^{*}{\rm{COs}}\_{\rm{max}}}}$.

As the conversions of both HCOO^∗^ and ^∗^CO_s_ require the presence of ^∗^H (absorbed hydrogen) involved^24^, *r*_CH4_ (${r_{{\,}^{*}{\rm{COs}}\_{\rm{max}}}}$) is determined by the concentrations of ^∗^CO_s_ ([^∗^CO_s_]) and ^∗^H ([^∗^H]), as well as the rate constant of ^∗^CO_s_ conversion (*k*_1_):

2 $${r_{{\rm{CH}}4}} = {r{*}_{{\rm{COs}}\_{\rm{max}}}} = {k_1}\left[ {\,}^{*}{{\rm{C}}{{\rm{O}}_{\rm{s}}}} \right]\left[ {\,}^{*}{{\rm{H}}} \right]$$

and ${r_{{\rm{HCOO}}^{*}}}$ is determined by the concentration of HCOO^∗^ ([HCOO^∗^]) and ^∗^H ([^∗^H]), and rate constant of HCOO^∗^ reduction (*k*_2_):

3 $${r_{{\rm{HCOO}}}}{*} = {k_2}\left[ {{\rm{HCOO}}^{*}} \right]\left[{\,}^{*}{\rm{H}} \right],$$

so

4 $${r_{{\rm{CO}}}} = {r_{{\rm{HCOO}}}}{*} - {r_{{\rm{CH4}}}} = {r_{{\rm{HCOO}}}}{*} - {r{*}_{{\rm{COs}}\_{\rm{max}}}} \\ = {k_2}\left[ {\rm{HCOO}}^{*} \right]\left[ {{{{\,}^{*}\rm H}}} \right] - {k_1}\left[{\,}^{*}{{\rm CO}_{{\rm{s}}}} \right]\left[ {{{{\,}^{*}\rm H}}} \right]\hskip1.2pc$$

It is known that ^∗^CO_s_ and HCOO^∗^ do not share and compete for active sites, as they are located on Pd metal and Al_2_O_3_ support, respectively^22^. Therefore, [^∗^CO_s_] and [HCOO^∗^] can be independently changed to tune the rate of CO formation, *r*_CO_, as well as the reaction product distribution.

**Fig. 4:**
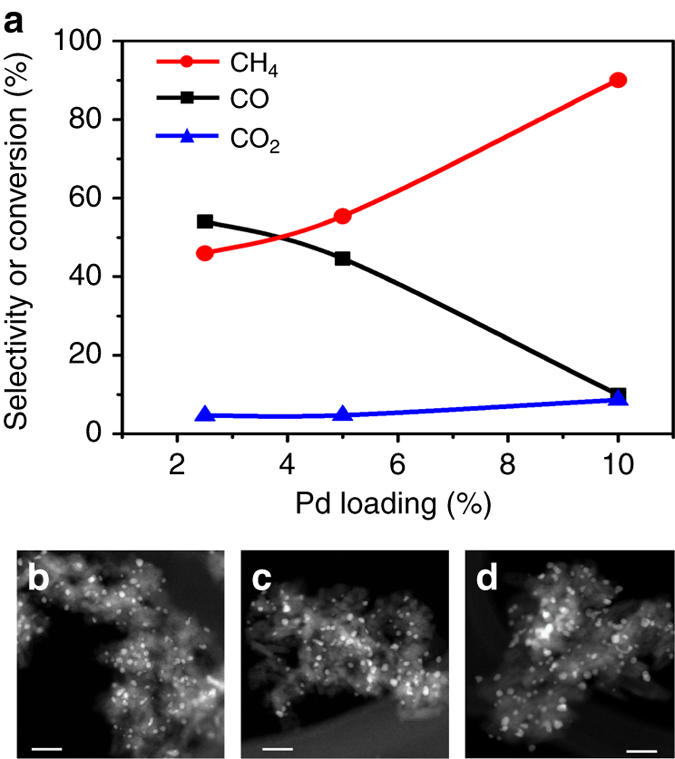
Controlled selectivities in CO_2_ reduction by tailor-made catalysts through mechanistic understanding. **a** CH_4_ and CO selectivities and CO_2_ conversion as a function of Pd loading at 573 K. **b**–**d** STEM images of 2.5, 5 and 10% Pd/Al_2_O_3_. STEM scale bars represent 20 nm. (Particle size distributions and temperature-programed ^∗^^12^CO desorption for the catalysts are displayed in Supplementary Fig. 8)

In the case of a completely filled pool of ^∗^CO_s_ (e.g., the case in Fig. 3), if aiming at a higher CH_4_ selectivity, [^∗^CO_s_] should be increased or [HCOO^∗^] should be decreased. One method, e.g., is to add more metal sites onto the Al_2_O_3_ support. The increased metal loading will not only result in an increased number of metal sites for forming ^∗^CO_s_ but also lead to a decreased number of support sites that can accommodate HCOO^∗^. It means that the capacity of ^∗^CO_s_ pool (Fig. 3) is enlarged, meanwhile that of HCOO^∗^ is decreased. This, consequently, may lead to a situation where the result of Eq. (4) decreases even to 0, showing less or complete absence of CO in the gas phase. This hypothesis was tested on Pd/Al_2_O_3_ catalysts with different Pd loadings but similar Pd particle size distributions (Fig. 4), which minimized the potential effects of metal particle size on *k*_1_ and *k*_2_ influencing ${r_{{\,}^{*}{\rm{COs}}\_{\rm{max}}}}$ and ${r_{{\rm{HCOO}}^{*}}}$. If the scheme and hypothesis are correct, Pd/Al_2_O_3_ catalysts with higher Pd loadings should exhibit higher CH_4_ selectivity than those with lower Pd loadings. Furthermore, this is exactly what the reactivity data of Fig. 4 shows: CH_4_ selectivity increased from 45 to 90% as the Pd loading was increased from 2.5 to 10% (Fig. 4). Previous studies on Ru/Al_2_O_3_ and Ni/SiO_2_ catalysts have also shown that CH_4_ selectivity in CO_2_ reduction reaction increased with the metal loading^12, 28, 29^, consistent with the findings reported in this work.

In the case of an incompletely filled pool of ^∗^CO_s,_ when CH_4_ selectivity is always 100%, if one aims at a higher CH_4_ formation rate, [^∗^CO_s_] needs to be increased. For instance, Karelovic et al.^30^ reported that the rate of CO_2_ methanation over Rh/Al_2_O_3_ was greatly increased by adding Pd/Al_2_O_3_, which has no activity towards CO_2_ methanation at 473 K. They attributed the synergistic effect to the supply of dissociated H^∗^ from Rh/Al_2_O_3_ to Pd/Al_2_O_3_. It is noteworthy that although CO_2_ methanation cannot proceed on Pd/Al_2_O_3_ at 473 K, this temperature is high enough for RWGS reaction to occur producing CO^22, 31^. We propose that CO or ^∗^CO produced by/on Pd/Al_2_O_3_ could diffuse onto the Rh domains, increasing the concentration of ^∗^CO_s_ on Rh. The total amount of ^∗^CO_s_ produced by both Pd/Al_2_O_3_ and Rh/Al_2_O_3_ is still not enough to saturate all the active Rh sites for CH_4_ formation (to fill up the ^∗^CO_s_ pool of Rh, Fig. 3). Therefore, the CH_4_ selectivity remained ~100% but the methanation rate was higher on the Pd/Al_2_O_3_-Rh/Al_2_O_3_ mixture than on Rh/Al_2_O_3_ alone. The role of the Pd/Al_2_O_3_ component was to provide extra CO (^∗^CO) to the empty sites of Rh. The synergistic effect at lower catalyst temperature (423 K) was found to be negligible. Our study showed that RWGS cannot occur over Pd/Al_2_O_3_ at 423 K^31^, so the added Pd/Al_2_O_3_ cannot supply additional CO to Rh/Al_2_O_3_ catalyst for the further ^∗^CO hydrogenation to CH_4_ on Rh/Al_2_O_3_. Therefore, the above analysis of their results show that for an incompletely filled pool of ^∗^CO_s_ with 100% CH_4_ selectivity (${r_{{\rm{HCOO}}^{*}}}$ ≤ ${r_{{\,}^{*}{\rm{COs}}\_{\rm{max}}}}$) on a catalyst, adding a component (promoter) which can produce CO (^∗^CO) is a strategy for increasing the rate of CO_2_ methanation.

In conclusion, CO_2_ methanation and RWGS are not two parallel reactions during the CO_2_ reduction over Pd/Al_2_O_3_ catalysts. Instead, they share the initial steps and intermediates from bicarbonates to formates until after formate decomposition. The rate of formate decomposition to CO^∗^ is larger than the rate of ^∗^CO hydrogenation to CH_4_ and the excess CO^∗^ desorbs. The rate-determining step for CO_2_ reduction and for RWGS is the conversion of HCOO^∗^, whereas that for CH_4_ formation is the hydrogenation of ^∗^CO. The balance of the hydrogenation kinetics between HCOO^∗^ and ^∗^CO governs the selectivities to CH_4_ and CO. Given that ^∗^CO and HCOO^∗^ are mainly on metal (Pd) and support (Al_2_O_3_), respectively, the balance could be tuned to achieve the desired CH_4_ and CO selectivities by optimizing the loading of the metal and the surface area of the support. This work has important implications for other bifunctional systems where the balance between different catalytic functions determines the rates and product distribution.

## Methods

### Catalyst synthesis and SSITKA experiments

The Pd/Al_2_O_3_ catalysts were prepared on a γ-Al_2_O_3_ powder (Sasol, Puralox SBA-200) by the incipient wetness method using Pd(NH_3_)_4_(NO_3_)_2_ as the precursor. After impregnation, the samples were dried at 373 K for 24 h and then calcined at 773 K for 2 h in air (flow rate = 60 mL min^−1^) and followed by reduction at 773 K for 1 h in 10% H_2_/He (flow rate = 60 mL min^−1^) to obtain the Pd/Al_2_O_3_ catalysts^31^. Forty-one milligrams of 5 wt% Pd/Al_2_O_3_ was pressed onto a tungsten mesh and loaded into a home-made *operando* transmission IR cell^32^. The cell has a very small internal dead volume (~0.2 cm^3^ after the catalyst loading), resulting in a short gas hold-up time. This renders the system suitable for obtaining accurate kinetic information about intermediates and products during the SSITKA experiments. Before experiments, the catalyst was pretreated by calcination at 673 K for 1 h under air with a flow rate of 10 mL min^−1^, followed by reduction at 673 K for 1 h under 20% H_2_ in He with a flow rate of 10 mL min^−1^. The reactant gas mixture was composed of 4 mL min^−1^ H_2_, 1 mL min^−1 12^CO_2_ or ^13^CO_2_, 1 mL min^−1^ Ar and He as the diluent with a total gas flow of 10 mL min^−1^ at atmospheric pressure. Ar gas was used as an inert tracer to correct for gas hold-up of the system and for gas re-adsorption. After the reaction reached steady state at the reaction temperature of 533 K, the reactant was switched from H_2_/^12^CO_2_/Ar to H_2_/^13^CO_2_. During the switch, the gaseous effluent from the cell and the species on the catalyst surface were monitored by MS and FTIR, respectively. The switch was performed in the temperature range of 533–573 K, where the CO_2_ conversion was <8%. In order to obtain the real mean surface residence times (${{{ \bar \tau }}_{{0}}}$) of intermediates leading to CH_4_ and CO, space velocity experiments were conducted by varying the total flow rate from 10 to 25 mL min^−1^ at constant partial pressures of CO_2_ and H_2_ to exclude gas hold-up and re-adsorption effects.

### Data analysis

The SSITKA theory has earlier been described extensively^16, 33^. The integration of the normalized step-decay or step-input response always yields the overall mean surface-residence time, ${{ \bar \tau }}$, of all adsorbed surface intermediates, which lead to CH_4_ and CO products $\left( {{{ \bar \tau }} = {\int}_0^\infty {F(t){\rm{d}}t} } \right)$. The error <5% caused by gas phase hold-up (~3 s) could be ignored due to the fast decay of standard Ar gas and CO_2_ gas. The rate constant is simply the reciprocal of average surface residence time for the active surface intermediates (*k* = ${{{ \bar \tau }}^{ - {\rm{1}}}}$ = TOF^∗^ *θ* ^−1^). The number of adsorbed species $\left( {{{{{ \bar N}}}_{{{\rm ICH}}}}} \right)$ and CO $\left( {{{{{ \bar N}}}_{{{\rm ICO}}}}} \right)$ can be obtained from integration of decay rates (*r*) of CH_4_ and CO products $\left( {\bar N = {\int}_0^\infty {r(t){\rm{d}}t} } \right)$. In addition, ${{ \bar N}}$ is usually closely related to the number of active sites on the catalysts for product formation. In this study of CO_2_ reduction over 41 mg of 5% Pd/Al_2_O_3_, the ${{{ \bar N}}_{{{\rm ICO}}}}$ completely originates from H^12^COO^∗^, which was demonstrated to be located on the Al_2_O_3_ support. As not all H^12^COO^∗^ was converted to ^12^CO, the amount of sites for formates on Al_2_O_3_ is estimated to be at least 1.86 μmol, i.e., ${{{ \bar N}}_{{{\rm ICO}}}}$ obtained at 533 K. For CH_4_ formation via ^∗^^12^CO_s_ and a portion of H^12^COO^∗^, the upper limit of the amount of active Pd sites for ^∗^CO_s_ (forming CH_4_) is 1.95 μmol, i.e., ${{{ \bar N}}_{{{\rm ICH}}}}$ at 533 K. This value accounts for 90% of the surface Pd atoms. The total amount of intermediates (${{{ \bar N}}_{{{\rm CO}}}}$ + ${{{ \bar N}}_{{{\rm CH4}}}}$) on the surface of the catalyst slightly decreased from 3.81 μmol at 533 K to 3.38 μmol at 573 K, which was caused by the decreased amount of H^12^COO^∗^ on the surface shown in Supplementary Fig. 7.

### Data availability

The data that support the findings of this study are available from the corresponding author on request.

## Electronic supplementary material

Supplementary Information
