# Supplementary material for: Controlling selectivities in CO2 reduction through mechanistic understanding
Source: Nat Commun. 2017 Sep 11;8:513. doi: 10.1038/s41467-017-00558-9 (PMC5594010; doi:10.1038/s41467-017-00558-9)
Supplement: Supplementary file 1 — Supplementary Information [file 41467_2017_558_MOESM1_ESM.pdf]

Description of Supplementary Files

File Name: Supplementary Information
Description: Supplementary Figures and Supplementary Table

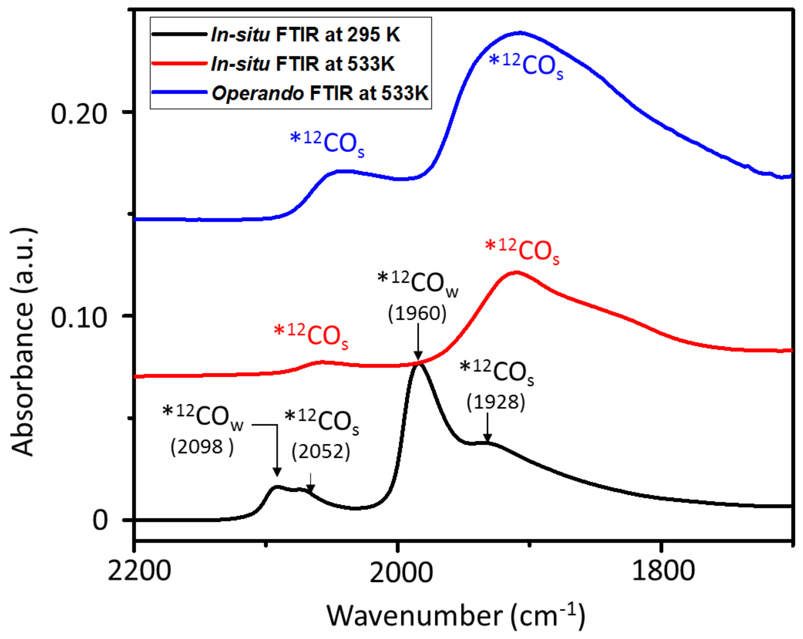

Supplementary Figure 1. In-situ FTIR spectra of CO adsorption on 5% Pd/Al_2O_3 at 295 K (Black), and then heated up to 533 K (Red), and *operando* FTIR spectrum (Blue) of 5% Pd/Al_2O_3 collected at 533 K in CO_2+H_2 reaction condition.

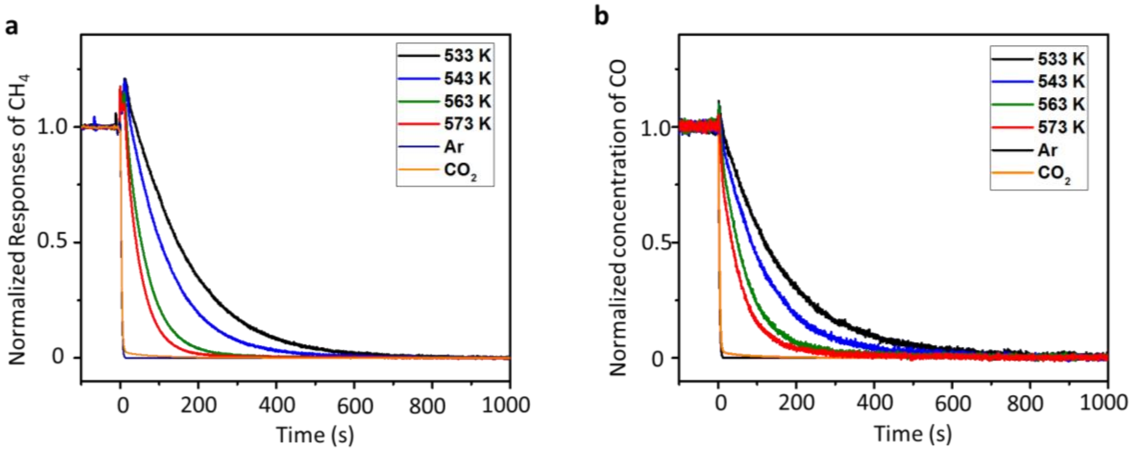

Supplementary Figure 2. Normalized MS signals of CH_4 (a) and CO (b) obtained at the exit of the *operando*-FTIR cell as a function of time at 533-573 K when the feed gas was switched at 0 s from CO_2+H_2 to $^{13}CO_2$+H_2 over 5% Pd/Al_2O_3.

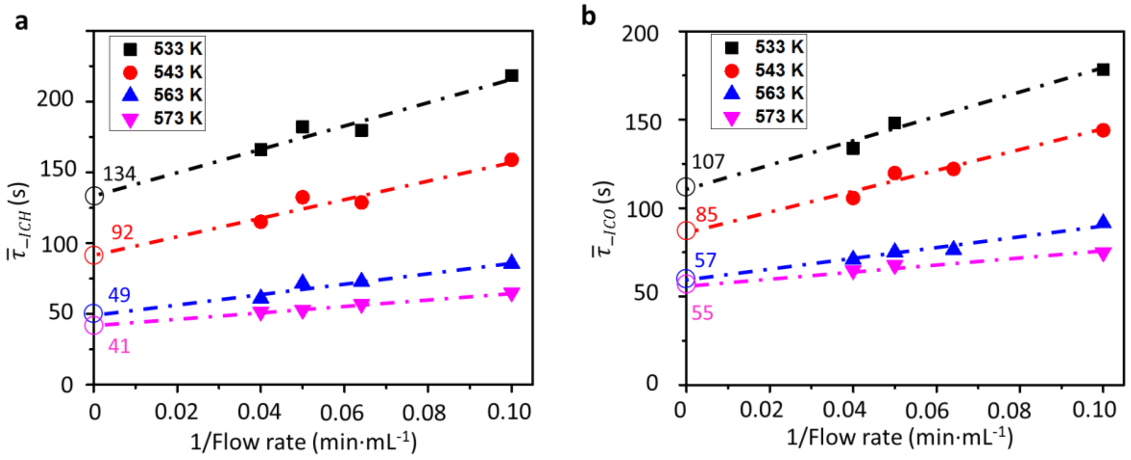

Supplementary Figure 3. Real mean surface residence times ($\bar{\tau}_0$) of ICH (a) and ICO (b) at 533-573 K obtained by extrapolating the mean surface residence times ($\bar{\tau}$) at different contact times of 0.04, 0.05, 0.67 and 0.1 min•mL^{-1}(by varying space velocity) to zero contact time. The mean surface residence time ($\bar{\tau}$) is yielded by integration of the normalized step-decay response of adsorbed surface intermediate leading to CH_4 and CO (Fig. S2) after the feed gas was switched at 0 s from CO_2+H_2 to $^{13}CO_2$+H_2 over 5% Pd/Al_2O_3.

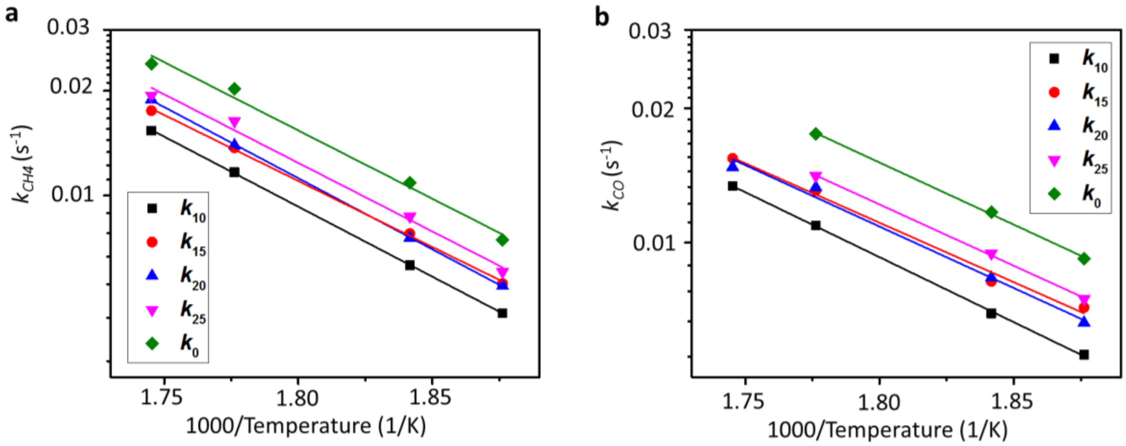

Supplementary Figure 4. Arrhenius plots for $^{12}CH_4$ (a) and ^{12}CO (b) formation at different contact times of 0 (k_0), 0.04 (k_{25}), 0.05 (k_{20}), 0.67 (k_{15}), 0.1 (k_{10}) min•mL^{-1}. The activation energies obtained from the plots are listed below in **Supplementary Table 1**.

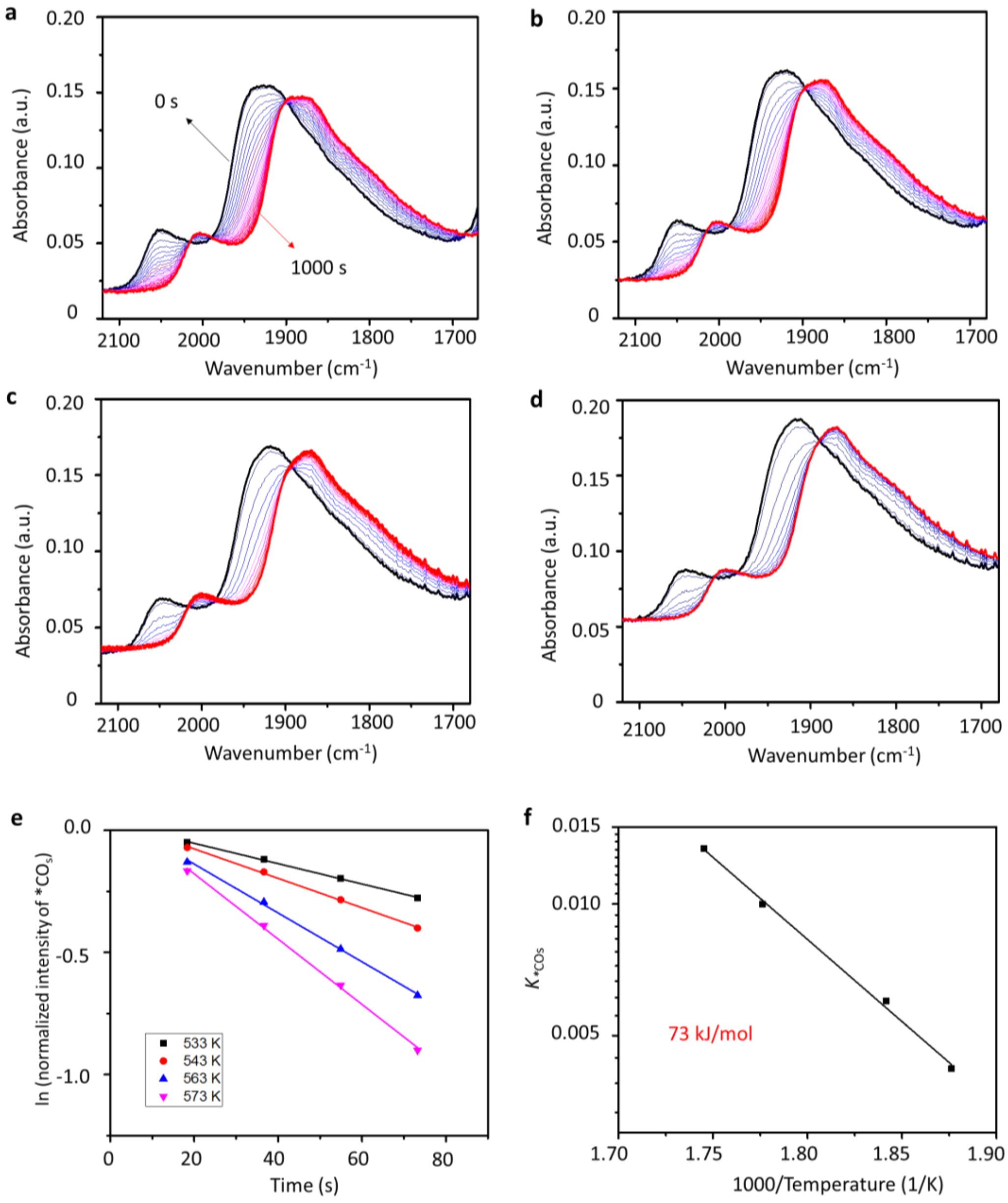

Supplementary Figure 5. Shift in peak positions of adsorbed $*^{12}CO_s$ to $*^{13}CO_s$ during *operando*-FTIR measurements on 5% Pd/Al_2O_3 at (a) 533, (b) 543, (c) 563 and (d) 573 K after the feed gas was switched at 0 s from $^{12}CO_2/H_2$ to $^{13}CO_2/H_2$. (e) The rate constants of adsorbed $*^{12}CO_s$ conversion at 533-573 K obtained by plotting the logarithm of the integrated band intensities (after peak deconvolution) versus time. (f) The activation energy for the conversion of $*^{12}CO_s$ obtained by fitting of reaction rates for the conversion of adsorbed $*^{12}CO_s$ from (e).

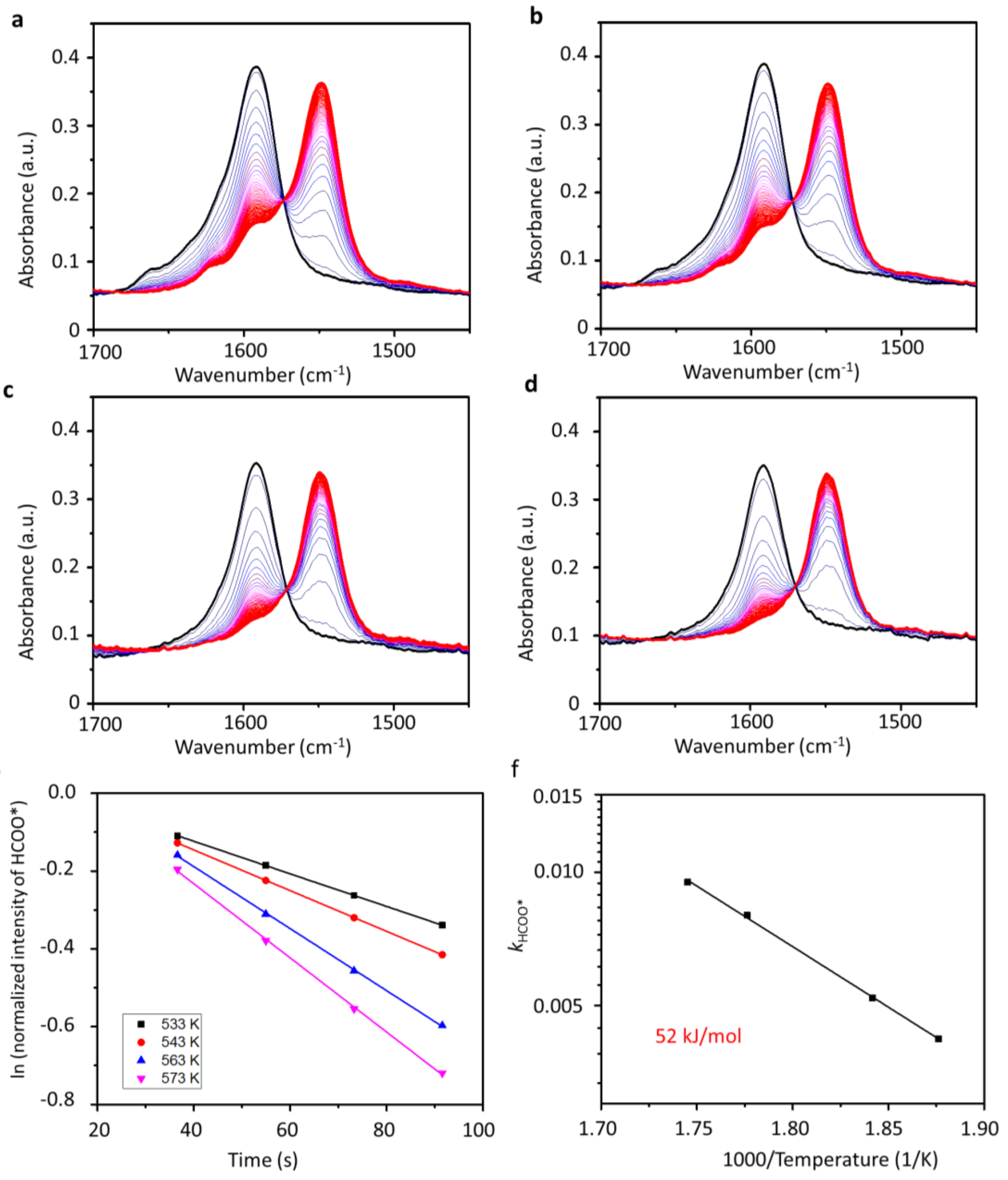

Supplementary Figure 6. Shift in peak positions of adsorbed $H^{12}COO*$ to $H^{13}COO*$ during *operando*-FTIR measurements on 5% Pd/Al_2O_3 at (a) 533, (b) 543, (c) 563 and (d) 573 K after the feed gas was switched at 0 s from $^{12}CO_2/H_2$ to $^{13}CO_2+H_2$. (e) The rate constants of adsorbed $H^{12}COO*$ conversion at 533-573 K obtained by plotting the logarithm of the integrated band intensities (after peak deconvolution) versus time. (f) The activation energy for the conversion of $H^{12}COO*$ obtained by fitting of reaction rates for the conversion of adsorbed $H^{12}COO*$ from (e).

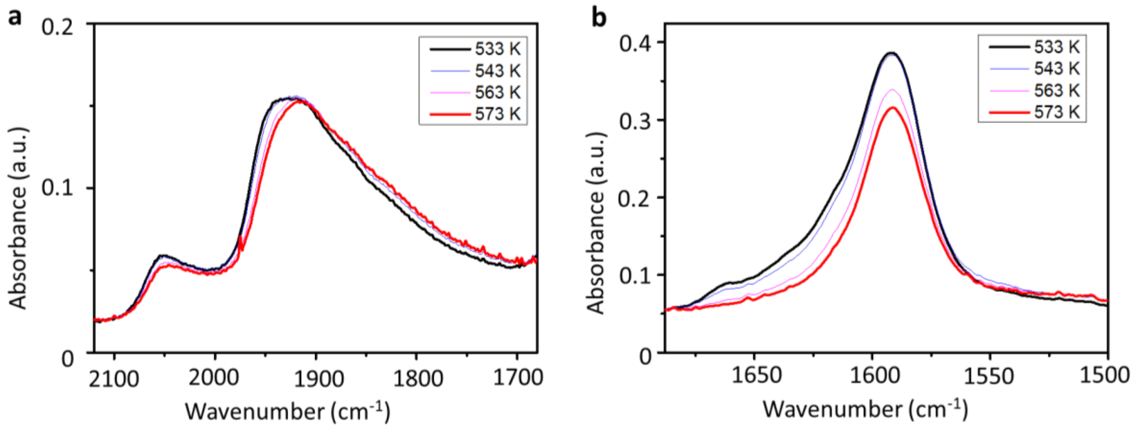

Supplementary Figure 7. *Operando*-FTIR spectra for adsorbed (a) $*^{12}CO$ and (b) $H^{12}COO*$ on 5% Pd/Al_2O_3 at 533, 543, 563 and 573 K obtained under steady state reaction conditions. (The size of the circle in Fig. 2 qualitatively corresponds to the amount of surface formates, deduced from the IR intensities in (b))

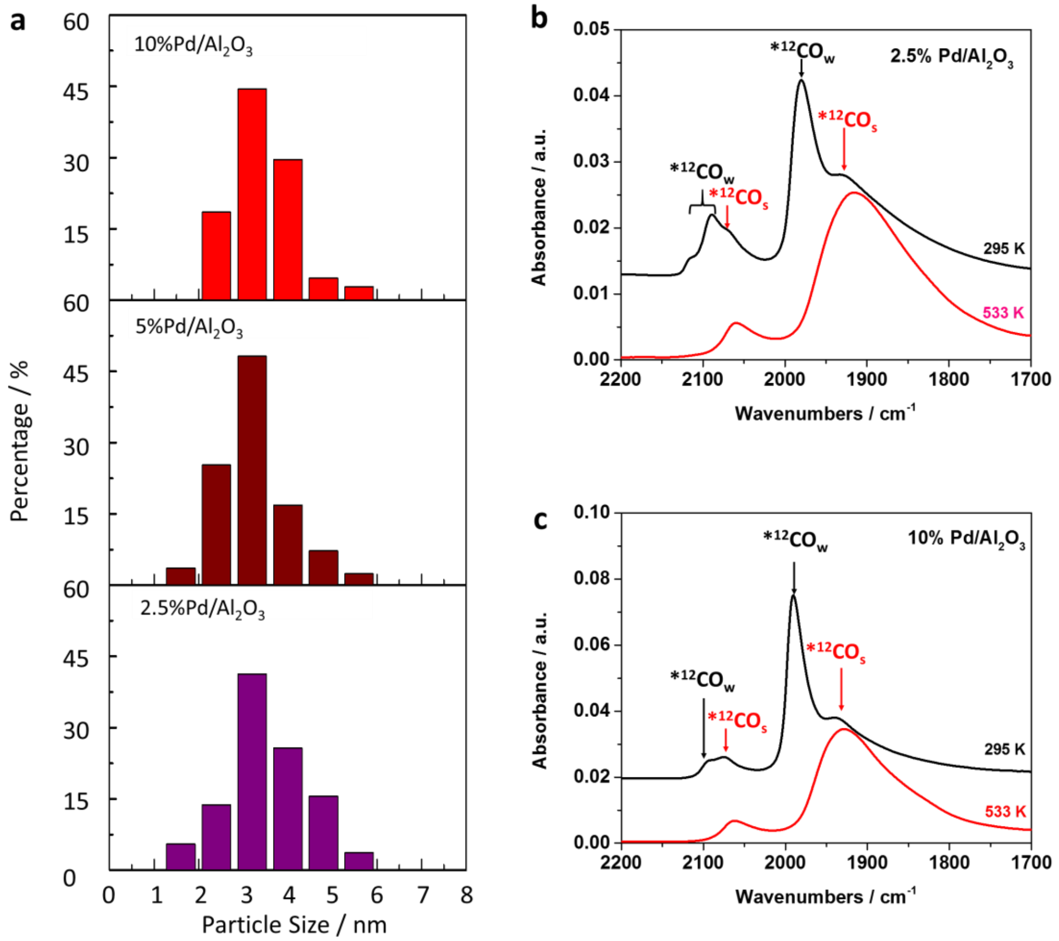

Supplementary Figure 8. (a) Pd particle size distribution analyzed from TEM images of 10% Pd/Al_2O_3, 5% Pd/Al_2O_3 and 2.5% Pd/Al_2O_3 (minimum number of particles analyzed in each histogram is 500). (b) In-situ FTIR spectra of CO adsorption on (b) 2.5% Pd/Al_2O_3 and (c) 10%Pd/Al_2O_3 at 295 K (Black), and then heated up to 533 K (Red).

Supplementary Table. The activation energies obtained from the plots in Supplementary Fig. 4.

	**Contact time (min/mL)**				
	**0.1 (k_{10})**	**0.67 (k_{15})**	**0.05 (k_{20})**	**0.04 (k_{25})**	**0 (k_0)**
**E_{CH4} (kJ/mol)**	77	72	78	75	75
**E_{CO} (kJ/mol)**	56	51	53	53	53
